# Supplementary material for: Predicting visual function by interpreting a neuronal wiring diagram
Source: Nature. 2024 Oct 2;634(8032):113–23. doi: 10.1038/s41586-024-07953-5 (PMC11446822; doi:10.1038/s41586-024-07953-5)
Supplement: Supplementary file 6 — CRF and ERF predictions for individual TmY4 and TmY9 cells. Analogous to Supplementary Data 3, but for TmY target types. Shown are the top four monosynaptic pathways, the strongest pathway passing through each of the top ten intermediary types (ranking from Extended Data Fig. 7), and the trisynaptic pathway Tm1–TmY–Dm3–TmY (see the section entitled Prediction of spatial normalization). [file 41586_2024_7953_MOESM6_ESM.zip › DataS4/TmY9qΓèÑ/720575940617573777.pdf]

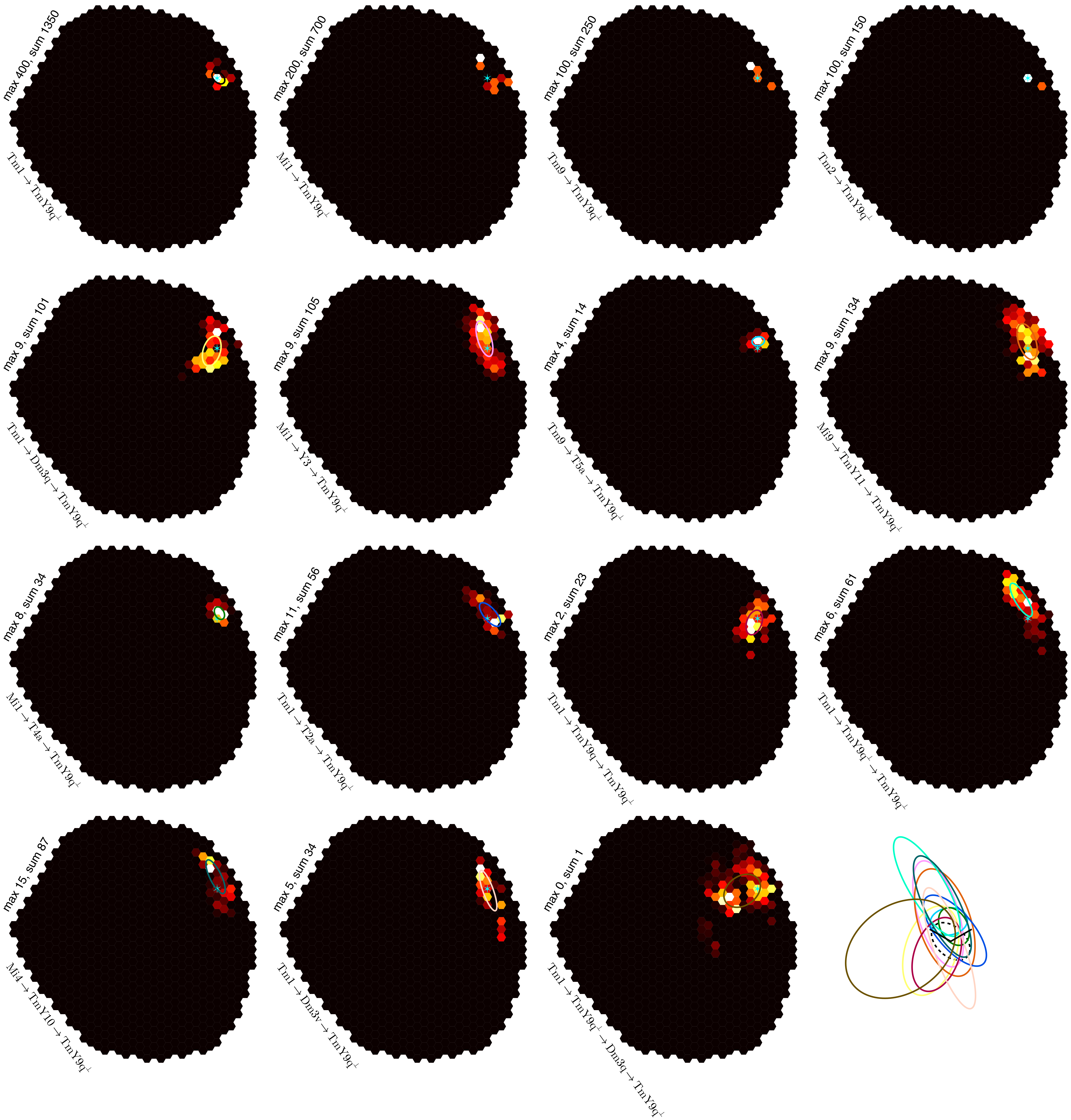

max 400, sum 1350
Tm1 → TmY9q⊥
max 200, sum 700
Mi1 → TmY9q⊥
max 100, sum 250
Tm9 → TmY9q⊥
max 100, sum 150
Tm2 → TmY9q⊥
max 9, sum 101
Tm1 → Dm3q → TmY9q⊥
max 9, sum 105
Mi1 → Y3 → TmY9q⊥
max 4, sum 14
Tm9 → T5a → TmY9q⊥
max 9, sum 134
Mi9 → TmY11 → TmY9q⊥
max 8, sum 34
Mi1 → T4a → TmY9q⊥
max 11, sum 56
Tm1 → T2a → TmY9q⊥
max 2, sum 23
Tm1 → TmY9q → TmY9q⊥
max 6, sum 61
Tm1 → TmY9q⊥ → TmY9q⊥
max 15, sum 87
Mi4 → TmY10 → TmY9q⊥
max 5, sum 34
Tm1 → Dm3v → TmY9q⊥
max 0, sum 1
Tm1 → TmY9q⊥ → Dm3q → TmY9q⊥
